# Supplementary material for: Causal Relationship between Aging and Anorexia Nervosa: A White-Matter-Microstructure-Mediated Mendelian Randomization Analysis
Source: Biomedicines. 2024 Aug 16;12(8):1874. doi: 10.3390/biomedicines12081874 (PMC11351342; doi:10.3390/biomedicines12081874)
Supplement: Supplementary file 1 [file biomedicines-12-01874-s001.zip › Supplementary Figure/Figure S4/Singe SNP plot of MR analysis for TL to ubm-b-1699.pdf]

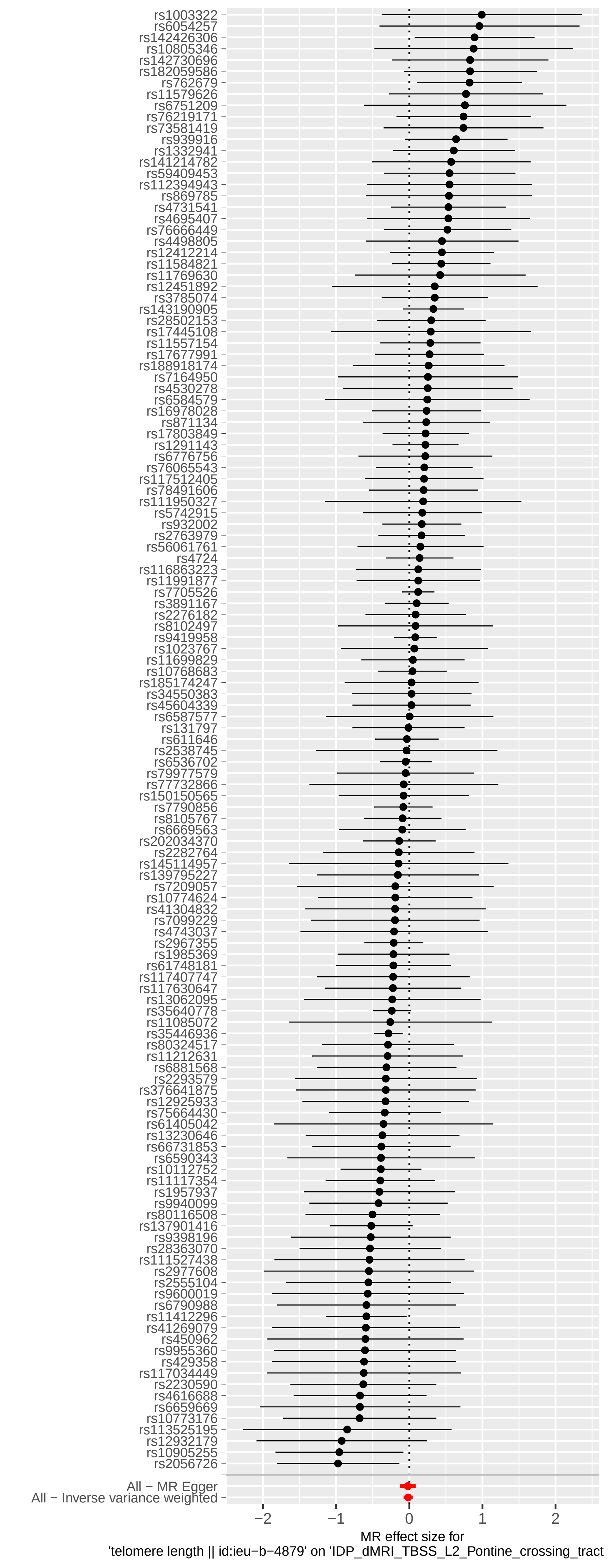
rs1003322
rs6054257
rs142426306
rs10805346
rs142730696
rs182059586
rs762679
rs11579626
rs6751209
rs76219171
rs73581419
rs939916
rs1332941
rs141214782
rs59409453
rs112394943
rs869785
rs4731541
rs4695407
rs76666449
rs4498805
rs12412214
rs11584821
rs11769630
rs12451892
rs3785074
rs143190905
rs28502153
rs17445108
rs11557154
rs17677991
rs188918174
rs7164950
rs4530278
rs6584579
rs16978028
rs871134
rs17803849
rs1291143
rs6776756
rs76065543
rs117512405
rs78491606
rs111950327
rs5742915
rs932002
rs2763979
rs56061761
rs4724
rs116863223
rs11991877
rs7705526
rs3891167
rs2276182
rs8102497
rs9419958
rs1023767
rs11699829
rs10768683
rs185174247
rs34550383
rs45604339
rs6587577
rs131797
rs611646
rs2538745
rs6536702
rs79977579
rs77732866
rs150150565
rs7790856
rs8105767
rs6669563
rs202034370
rs2282764
rs145114957
rs139795227
rs7209057
rs10774624
rs41304832
rs7099229
rs4743037
rs2967355
rs1985369
rs61748181
rs117407747
rs117630647
rs13062095
rs35640778
rs11085072
rs35446936
rs80324517
rs11212631
rs6881568
rs2293579
rs376641875
rs12925933
rs75664430
rs61405042
rs13230646
rs66731853
rs6590343
rs10112752
rs11117354
rs1957937
rs9940099
rs80116508
rs137901416
rs9398196
rs28363070
rs111527438
rs2977608
rs2555104
rs9600019
rs6790988
rs11412296
rs41269079
rs450962
rs9955360
rs429358
rs117034449
rs2230590
rs4616688
rs6659669
rs10773176
rs113525195
rs12932179
rs10905255
rs2056726
All − MR Egger
All − Inverse variance weighted
−2
−1
0
1
2
MR effect size for
'telomere length || id:ieu−b−4879' on 'IDP_dMRI_TBSS_L2_Pontine_crossing_tract
